# A Mobile Sensor Network System for Monitoring of Unfriendly Environments

**DOI:** 10.3390/s8117259

**Published:** 2008-11-14

**Authors:** Guangming Song, Yaoxin Zhou, Fei Ding, Aiguo Song

**Affiliations:** School of Instrument Science and Engineering, Southeast University, Nanjing, 210096, P.R. China; E-Mails: bluesensor@gmail.com; robotsensor@126.com; a.g.song@seu.edu.cn

**Keywords:** Wireless sensor network, Mobile sensor network, Mobile node, Localization, Deployment

## Abstract

Observing microclimate changes is one of the most popular applications of wireless sensor networks. However, some target environments are often too dangerous or inaccessible to humans or large robots and there are many challenges for deploying and maintaining wireless sensor networks in those unfriendly environments. This paper presents a mobile sensor network system for solving this problem. The system architecture, the mobile node design, the basic behaviors and advanced network capabilities have been investigated respectively. A wheel-based robotic node architecture is proposed here that can add controlled mobility to wireless sensor networks. A testbed including some prototype nodes has also been created for validating the basic functions of the proposed mobile sensor network system. Motion performance tests have been done to get the positioning errors and power consumption model of the mobile nodes. Results of the autonomous deployment experiment show that the mobile nodes can be distributed evenly into the previously unknown environments. It provides powerful support for network deployment and maintenance and can ensure that the sensor network will work properly in unfriendly environments.

## 1. Introduction

Wireless sensor networks are changing our way of life just as the Internet has revolutionized the way people communicate with each other. Wireless sensor networks combine distributed sensing, computation and wireless communication. This new technology expands our sensing capabilities by connecting the physical world to the communication networks and enables a broad range of applications. Observing microclimate changes is one of the most popular applications of wireless sensor networks [1]. Sensor nodes can be deeply embedded and densely deployed to enable up-close monitoring of various indoor or outdoor environments. However, some environments are often too dangerous or inaccessible to humans. For example, a building on fire or a suspected hazardous material leak. Although monitoring of sensitive wildlife and habitats has few potential hazards, the intrusion of humans is always a bothersome problem. Some environments cannot be by humans or large robots because of terrain and space limitations. In all these situations, wireless sensor network users will face many challenges, such as deployment, network maintenance and repair.

In recent years, the interaction of distributed robotics and wireless sensor networks has led to the creation of mobile sensor networks. It is considered that augmenting static sensor networks with mobile nodes can solve many of the research challenges that exist in static sensor networks [2]. A mobile sensor network is composed of a distributed collection of enhanced nodes. Each node has sensing, computation, communication and locomotion modules. Compared to the conventional static wireless sensor networks, mobile sensor networks have more powerful network capabilities such as self-deployment, network repair and event tracking. Each mobile senor node is capable of navigating autonomously or under control of humans. Large numbers of mobile sensor nodes can coordinate their actions through ad-hoc communication networks.

Some research groups have begun to design mobile nodes for wireless senor networks and have made some prototypes. In [3], The Robomote is introduced as a tabletop platform for experiments on mobile sensor networks. It is more than 1,300 times smaller than Pioneer robots which are commonly used in laboratories across the world. CotsBots is another modular robot platform for research in distributed robotics [4]. It is built entirely from off-the-shelf components and requires minimal assembly. Some additional sensor modules need to be added to the CotsBots platform in order to get better performance in a sensor network environment. Other similar platforms such as Millibots, MICAbot and Racemote also have met the concept of mobile sensing [5-9].

Although the existing platforms have provided initial support for developing large-scale mobile sensor networks and distributed robotics, their controllability still needs improvement. How to build models for mobility and networking management and how to interact with these special networked systems still need to be further investigated. This paper presents a mobile sensor network system for monitoring of unfriendly environments. The system architecture, the mobile node design, the basic behaviors and advanced network capabilities have been investigated respectively. Details of this work will be presented in Section 2-4.

## 2. System Overview

**Figure 1.**
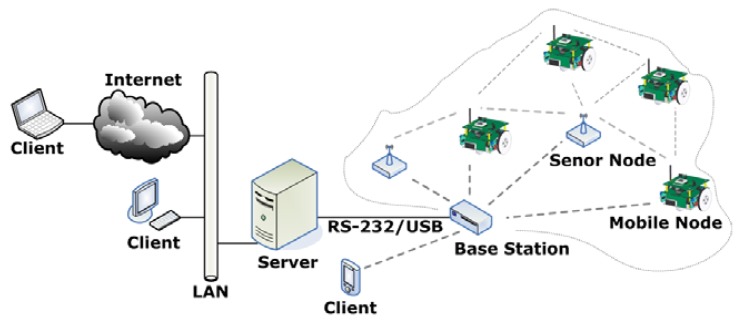
The system architecture of a mobile sensor network.

The complete system architecture of a mobile sensor network includes a group of mobile sensor nodes, a base station, upper communication network infrastructures and clients. As shown in Figure 1, the sensor nodes are scattered in the target environment and they form a multi-hop mesh networking architecture. Each of these sensor nodes has the capability of collecting data and routing data peer-to-peer to base stations. The mobile sensor node is in fact an enhanced sensor node. It not only has all the capabilities of the static sensor node, but also realizes mobility by adding a robotic base and a driver board. A base station is used to bridge the sensor network to another network or platform, such as Internet.

A mobile sensor network is well suited for distributed measurement and control applications. Its architecture can be divided into three layers: node layer, server layer and client layer. The node layer consists of all the sensor nodes that can be either static or mobile. This layer is directly embedded into the physical world to get all kinds of data. The server layer includes a personal computer or a single board computer running server software. The client layer includes local clients and remote clients. The devices of the client layer can be any smart terminals, such as PCs, PDAs, Pocket PCs and smart phones. The server layer and the client layer communicate with each other and they form a typical example of Internet [10].

## 3. Mobile Node Design

The so-called mobile sensor node is in fact a mobile robot that can communicate with other nodes wirelessly in the multi-hop sensor network. When we design a mobile node for wireless sensor networks, we can add various locomotion modules to the sensor nodes so that they can move from place to place. The differential drive robot is perhaps the simplest type of mobile robot [11]. Here we propose a wheel-based mobile node architecture that can be regarded as a simple differential drive robot. But we should notice that the wireless sensor node is a resource-constrained device. When we add mobility to it, we cannot expect it to be as powerful as conventional mobile robots, but often there is no need to make the mobile sensor nodes that powerful. For example, we can use a big robot to carry the mobile sensor nodes to the destination. When the big carrier robot encounters an obstruction in its path due to space and terrain limitations, the mobile senor nodes will be unloaded and they will continue by themselves.

### 3.1. Structure Decomposition

**Figure 2.**
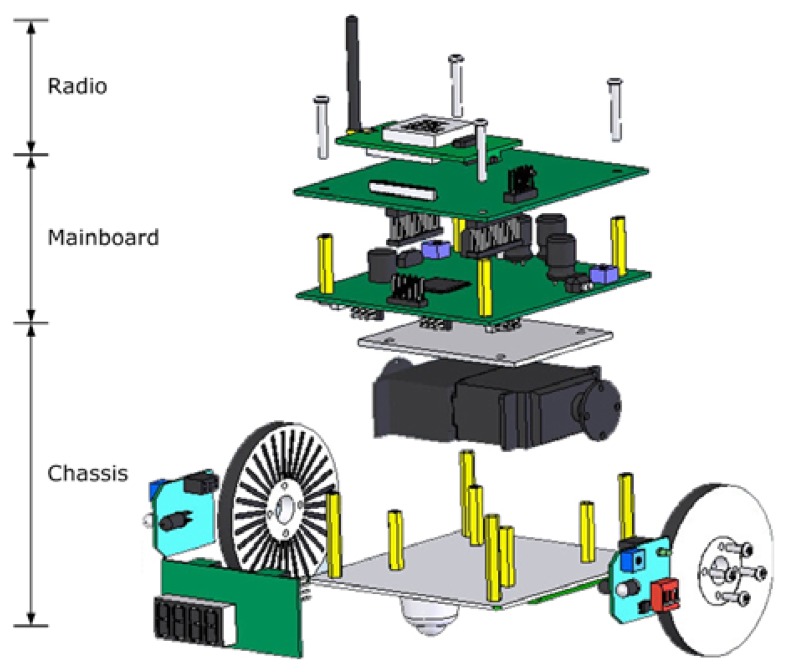
Exploded view of the proposed mobile node structure.

Figure 2 shows the structure of the proposed mobile sensor node in an exploded view. The whole architecture includes a sensor module, a radio module, a mainboard, a driver board and a chassis. The radio module is connected with the mainboard by an expansion connector. It works in the 2.4 GHz frequency band and establishes wireless communication channels with other nodes in range. The mainboard is the brain of this smart device. It is responsible for most of the data processing tasks inside the node and manages the wireless communication links to and from neighboring nodes. The chassis is the base platform custom designed for building our mobile sensor nodes. It has two separate driving wheels and one universal wheel for supporting the node body and flexible steering. A rechargeable NiMH battery pack is attached to the bottom of the chassis for power supply. A coprocessor is added to the mainboard for better performance on motor control and motion parameters detection. The sensor module is an optional module that varies from application to application. For example, in environment monitoring applications, the most commonly used sensors are temperature, humidity, light, atmospheric pressure and so on. In our design, we add two IR sensors to the front side of the mobile node for obstacle avoidance and add three light sensors to the mainboard for ambient lighting detection.

**Figure 3.**
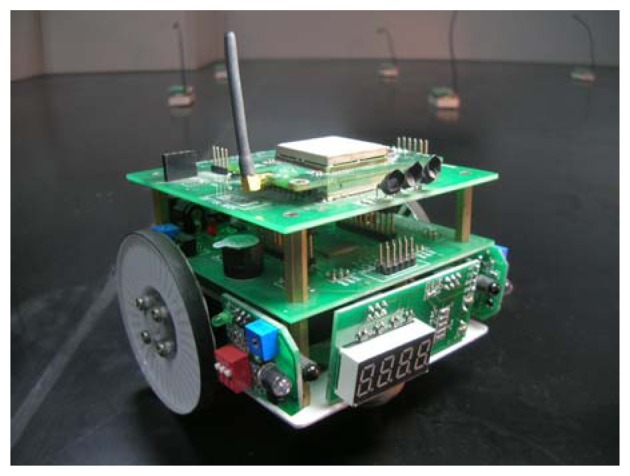
A prototype of the mobile node.

A prototype of the mobile sensor node we designed and implemented by following the proposed architecture is shown in Figure 3. The mobile node, which we call *RacemoteZ*, provides a novel robotic platform for adding controlled mobility to wireless sensor networks or other distributed measurement and control systems. The size of RacemoteZ is 105 mm×90 mm×80 mm. This tiny mobile node includes all the modules described above. Those modules are connected together via extended interfaces and they form a sandwich-like structure. By using this kind of structure, it is easy to assemble and disassemble the node. The extended interfaces make it possible to add additional modules to the node when a system upgrade is needed at a future date.

### 3.2. Node Networking

**Figure 4.**
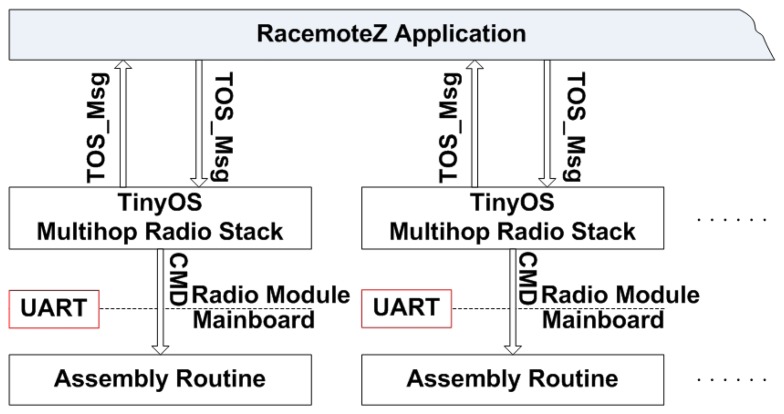
The embedded software modules of the mobile node.

The software environment for the sensor nodes is TinyOS, an open-source operating system designed for wireless embedded sensor networks [12]. The embedded software architecture of RacemoteZ is shown in Figure 4. A RacemoteZ communicates with other nodes or gateway by using a standard data format, namely *TOS_Msg*. The header of each TOS_Msg data packet that comes out of the radio module contains several data fields that are used to identify different network addresses, different network services and different network groups. The payload of TOS_Msg is interpreted as a command that will be sent to the mainboard to perform some actions, such as start, stop and move. If the node is performing a sensing task, the payload will be the ADC counts of the sensors. A RacemoteZ application receives command messages from the radio and interprets them. It will also forward command messages that it receives to other nodes in the network if necessary. This is accomplished by re-broadcasting the command message once it has been processed. In this way, a so-called *mesh network* is formed by following a multi-hop routing protocol.

### 3.3. Computing Models

**Figure 5.**
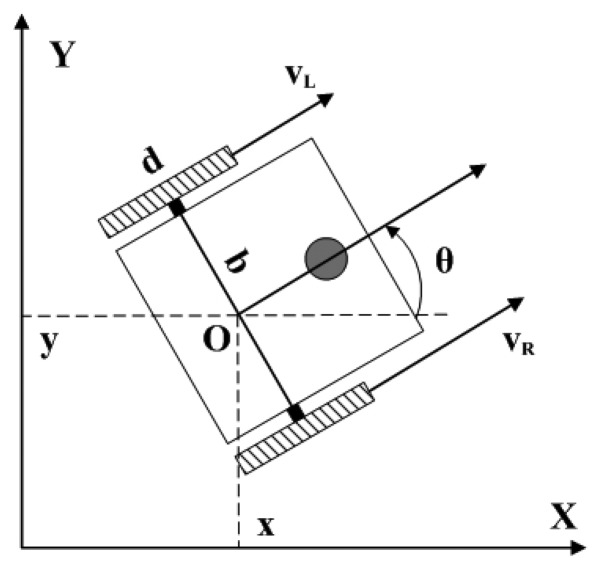
Computing model of the mobile node for 2D pose calculation.

Precisely localization of the position of the mobile node in unknown indoor environments is a big challenge. In those circumstances, GPS-based and beacon-based localization methods do not work well or even fail. Considering the size and cost limitations of the mobile node, it is feasible to use the dead reckoning method to estimate the node location. Although this method is well known for its large cumulative error, it is still a good choice here since the traveling distance of the mobile node is not always very long in most applications and the cumulative error will not exceed the acceptable limit. The computing model of the proposed mobile node for 2D pose estimation is shown in Figure 5. The pose of the mobile node can be expressed as:

(1) ξ=[x,y,θ]

where *O*(*x*, *y*) denotes the midpoint of the driving axle and *θ* denotes the orientation angle of the mobile node. When *v_L_* = -*v_R_*, the mobile node can acquire zero turning radius. Therefore the mobile node can reduce curvilinear motion to a great extent. It is helpful for the mobile node to improve its positioning accuracy. But due to machining and assembling errors, speed mismatch of the two motors and other errors, the actual trace of the mobile node can not be a straight line.

**Figure 6.**
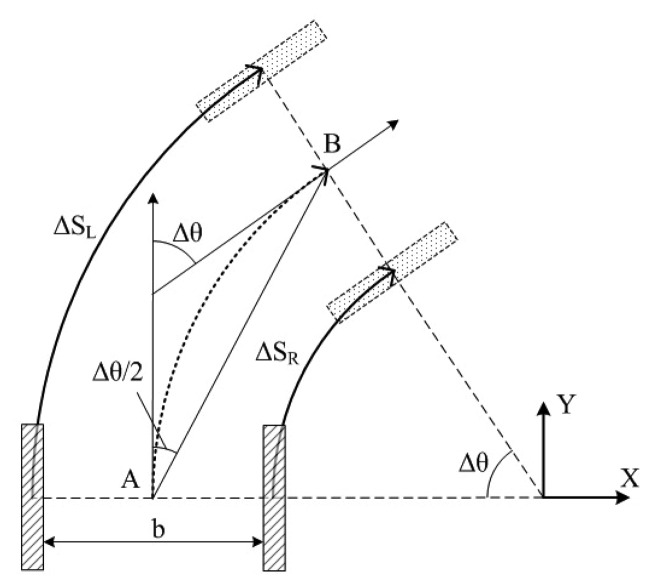
The principle of 2D pose calculation for the mobile node.

As shown in Figure 6, when the node moves from point *A* to point *B*, the trace between the two points is a curve *ΔS* If we control the sampling interval to be very short, *ΔS* can be considered to approximate to a straight line. Therefore the pose of the mobile node can be calculated as follows:

(2) $$\xi_{n+1}=\xi_{n}+\left[\begin{array}{c} \Delta S\cos(\theta +\Delta \theta /2)\\ \Delta S\sin(\theta +\Delta \theta /2)\\ \theta +\Delta \theta \end{array}\right]$$

where

(3) $$\Delta S(\Delta S_{L}+\Delta S_{R})/2$$

and *ΔS_L_* denotes the trace of the left wheel when the node moves from *A* to *B*, *ΔS_R_* denotes that of the right wheel, *Δθ* denotes the change in orientation and *b* denotes the track width.

**Figure 7.**
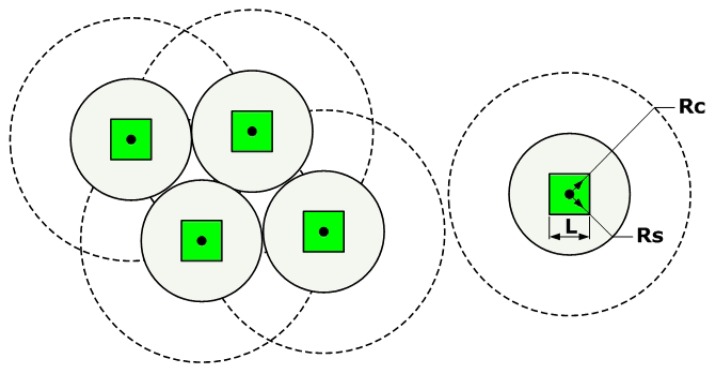
Sensing range and communicating range of the mobile node.

The sensing region of the mobile node is modeled as a circle, as shown in Figure 7. Its radius is *R_s_*. The communicating region of the mobile node is also modeled as a circle. Its radius is *R_c_*. In order to work properly, the mobile node needs to meet the following constraints:

(4) $$\frac{L}{\sqrt{2}}\leq R_{s}\leq \frac{R_{c}}{2}$$

Here the robotic chassis is approximately modeled as a square. Its side length is *L*. If $R_{s}=L/\sqrt{2}$ and the mobile nodes are deployed side by side, the total coverage area of the sensor network reaches its minimum value. If the distance between any two nodes is not shorter than 2*R_s_* and there is no obstacle in the covered region, then the total coverage area of the sensor network reaches its maximum value:

(5) $$A_{m}=N\pi R_{s}^{2}$$

where *N* denotes the number of nodes in the sensor network. Although the coverage area reaches its maximum value in this situation, we should notice that coverage holes appear among the sensor coverage circles.

## 4. Experiments

### 4.1. Testbed setup

**Figure 8.**
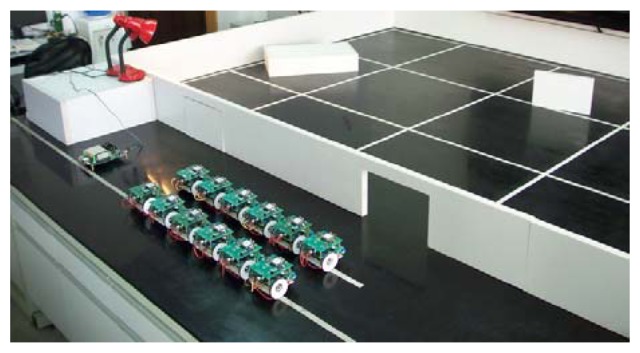
Testbed setup for the experiments on the mobile sensor network.

A testbed for validating the basic functions of the proposed mobile sensor network system has been created in our laboratory. As shown in Figure 8, a 2,800 mm × 2100 mm area with a rectangular enclosure is set aside on the flat surface of a tabletop testbed. The area is assumed to be inaccessible to human operators and the entrance is also too narrow for carrier robots to go inside and deploy sensor nodes there. Two different rectangular obstacles are placed on the surface to increase the complexity of the inside area. The infrastructure of wireless sensor network is established outside the enclosure which includes some sensor nodes and a base station. The mobile sensor nodes will depend on this network infrastructure to keep contact with the outside world when exploring in the so-called unknown environment.

### 4.2. Performance Tests

Before testing the networking behaviors of the mobile sensor nodes, we need to investigate their basic motion performance first. In the path planning of the mobile nodes, we use only two basic motion components, i.e. *go-straight* and *in-situ turning*. We have to measure the cumulative error of each basic motion respectively. Three mobile nodes are randomly selected from the group to perform the test. Each node is programmed to move on a flat table-top surface and perform only one basic motion at a time. Then the average errors are calculated to represent the accuracy of localization.

**Figure 9.**
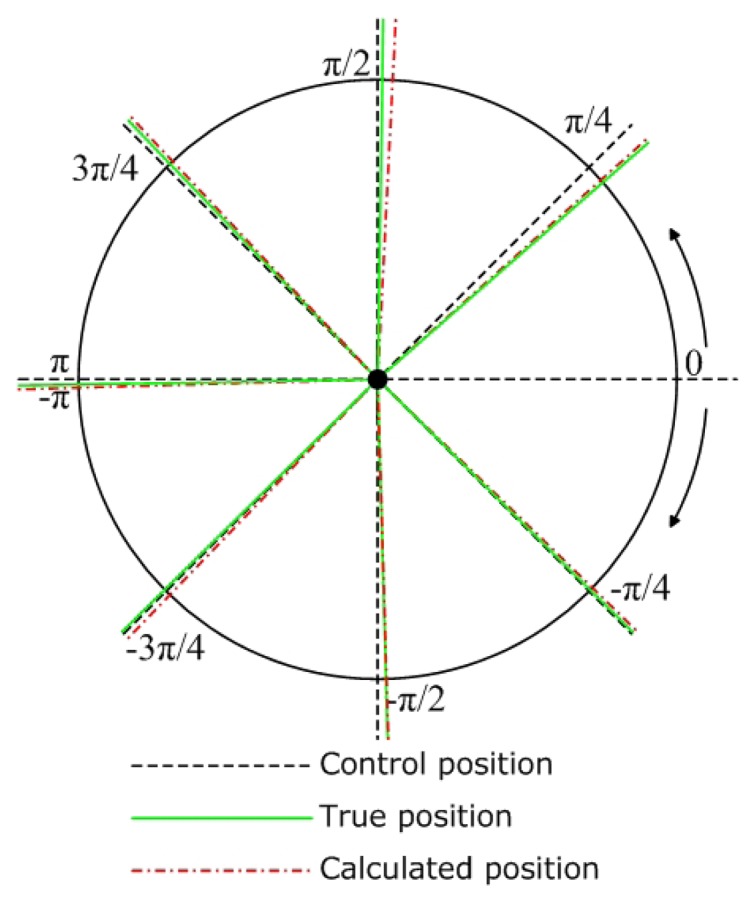
Cumulative errors in localization when the node performs in-situ turning.

**Figure 10.**
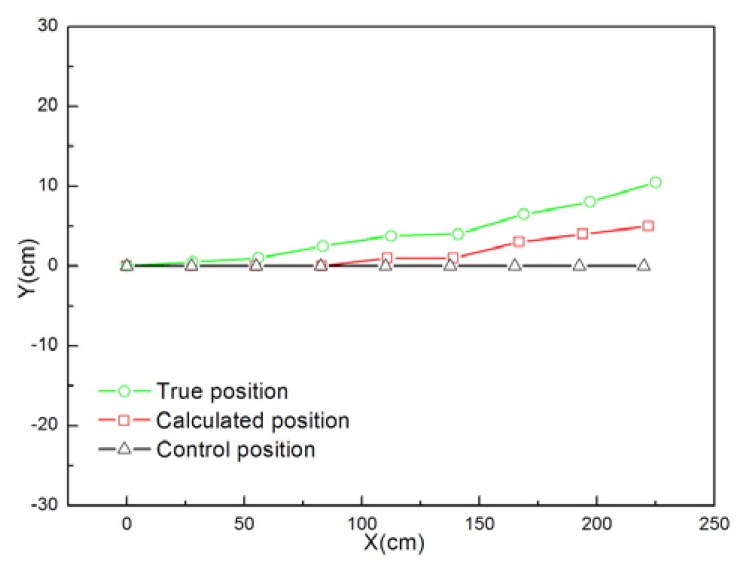
Cumulative errors in localization when the node is instructed to go straight.

Figures 9 and 10 show the cumulative error test results for mobile node localization. As shown in Figure 9, when the node performs in-situ turning, the calculated position is very close to the true position, but a steady deviation from the control position still exists. As shown in Figure 10, the node is programmed to move straight along the x-axis from the starting point (0, 0). After running for predefined steps it stops at the end point (2250, 105) and the calculated position of the end point is (2220, 50). The final trace of the mobile node shows that the positioning error increases faster in the y-direction. Those results were to a great extent caused by the errors of wheel diameter and track width. Although we can make compensation for the positioning errors according to the test results of each node, a more practical solution is to improve the accuracy of mechanical processing and assembly.

**Figure 11.**
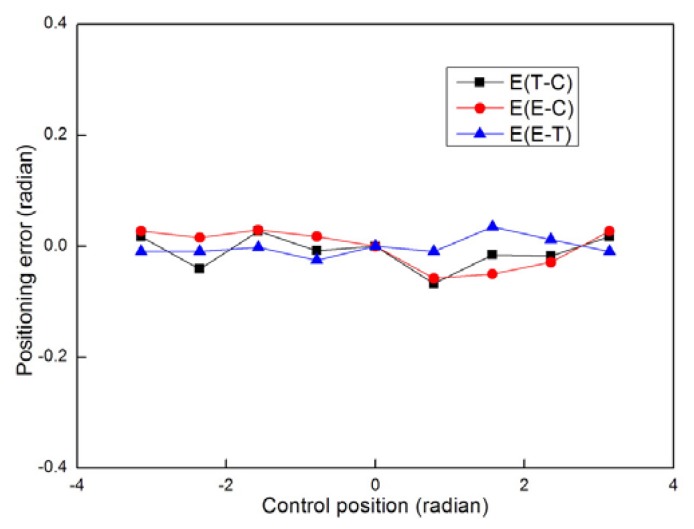
Error analysis of the in-situ turning test. E(T-C) denotes the error between true position and control position. E(E-C) denotes the error between calculated position and control position. E(E-T) denotes the error between calculated position and true position.

**Figure 12.**
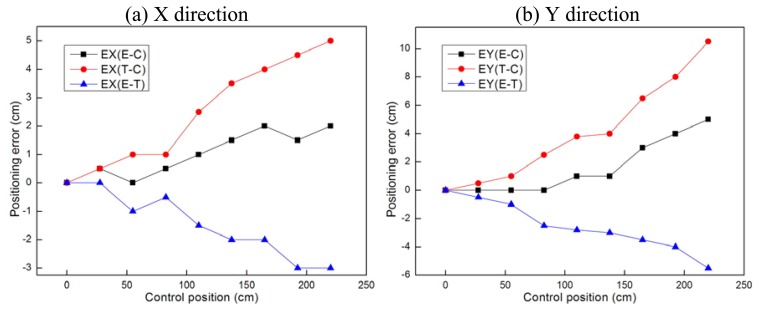
Error analysis of the straight-going test.

Figures 11 and 12 show the positioning error changes during the motion tests. According to the error curves in Figure 11, there is little change in the positioning errors during in-situ turning. According to the error curves in Figure 12, all three kinds of error distances increase with the travelling distance. Due to the size limitations of the testbed, we currently can only let the mobile node go a distance of no more than 3 meters. If the mobile node takes a longer route, we can deduce from the tests that the cumulative positioning errors will be too big to be acceptable for practical applications. Although we can make compensation or add more sensors to decrease the errors when the traveling distance is too long, we do not encourage others to do so. The primary design consideration of this kind of platform is that it should be as small and simple as possible. We do not expect it to be as powerful as the conventional mobile robots. It in fact serves as a small slave robot for nearby bigger robots or human operators. Therefore we should avoid forcing it to travel too far alone. We can use a big carrier robot to carry the mobile nodes to approach the target to the best of its abilities before the carrier robot can not move forward any more. And that will to a great extent decrease the distances the mobile nodes have to go.

EX(E-C) denotes the error between calculated position and control position in X direction. EX(T-C) denotes the error between true position and control position in X direction. EX(E-T) denotes the error between calculated position and true position in X direction. EY(E-C) denotes the error between calculated position and control position in Y direction. EY(T-C) denotes the error between true position and control position in Y direction. EY(E-T) denotes the error between calculated position and true position in Y direction.

In the above tests, we command the nodes to move at about eighty percent of its full speed. Since the speed of the two DC motors is regulated by Pulse Width Modulating, we directly use the PWM value to represent the motion speed. When we set the PWM value to its maximum value, the mobile node will reach a speed of 0.15m/s. The following tests aim to investigate what the performance of the node will be when varying the moving speed.

**Figure 13.**
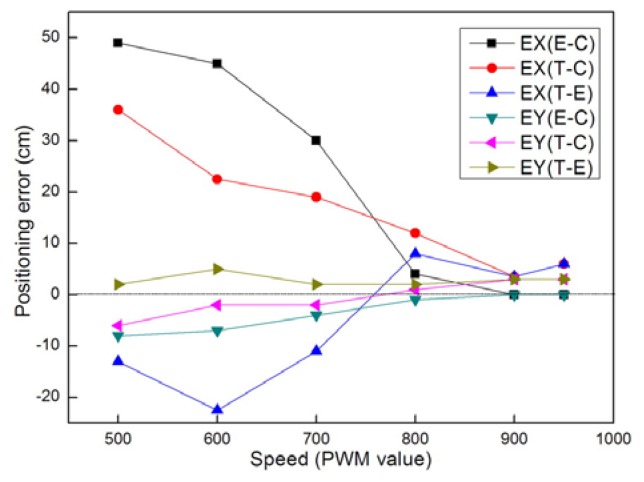
Positioning error changes when the node moves in different speed.

**Figure 14.**
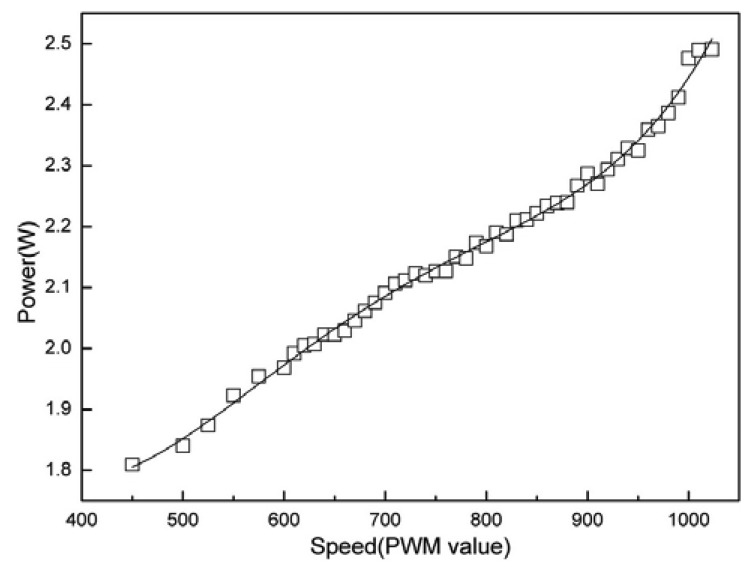
Power model of the mobile node.

Figure 13 shows the positioning error changes when the node moves in different speed. It can be seen that the positioning errors are relatively big when the speed is lower than 700 PWM value. But all the errors decrease sharply when the speed increases. So programming the node to move at a higher speed will help increase the localization accuracy. When the node moves in low speed, the driving forces output from the motors are also relatively small. So the influences of interior mechanical resistance and external friction will increase. When the speed is too low, those influences can even make either motor or both of them to stop working occasionally. That will cause the node to turn its direction unexpectedly when moving in low speed. So the positioning errors in X direction (orthogonal to the forwarding direction) are very big in the low speed scenario. The power model mainly concerns the relationship between speed and power. The mobile sensor node is powered by six 1.2V NiMH rechargeable batteries. We use two hand-held multimeters to measure the voltage and the current. The mobile sensor node is controlled to move straight at different speeds and the motion power is measured. During the process, the mobile node carries no additional load and moves on an indoor flat surface. The results of motion power measurement are shown in Figure 14. The power increases steadily and linearly as the speed increases. It gives us a reliable reference to judge whether the remaining power is enough to ensure the mobile node to finally reach its destination.

### 4.3. Autonomous Deployment

**Figure 15.**
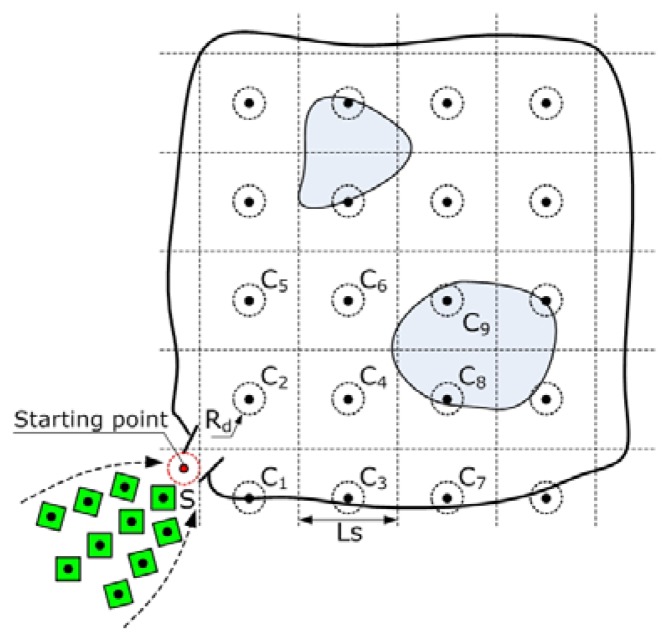
The principle of autonomous deployment of mobile sensor nodes.

How to deploy the mobile sensor nodes into the target environments is an important problem we need to tackle first, especially when they are going to be used in the so-called unfriendly environments [13-14]. Here we propose a sequenced grid based autonomous deployment method for our mobile sensor network system. We divide the region waiting to be covered into a number of small sections and deploy at least one mobile node into every section. In order to avoid coverage holes, we have to abandon the idea to pursue maximum coverage area. Instead, when the deployment task is completed, we expect the sensor coverage circles to lap over each other and the inscribed squares of every four neighboring circles are connected together to form a bigger square, as shown in Figure 15.

Then we can divide the target region into many identical square cells that connect with each other. The side length of each cell is $L_{s}=\sqrt{2}R_{s}$. We set a starting point *S* at the entrance and each mobile node has to start its deployment motion from this point. Each cell is then assigned to one of the mobile nodes and the end point is the centroid of each cell. Due to the positioning errors, the mobile nodes in fact can not precisely reach the starting point or the end points. So we have to add target circles to surround those points. The radius of the target circle *R_d_* is in proportion to the positioning accuracy of the mobile node.

We number the cells according to the distances between the starting point and the end points. The nearest cell to the starting point is assigned a sequence number one; the second nearest one is assigned a sequence number two, and so on. In order to improve the deployment efficiency and avoid unnecessary obstructions by the previously deployed nodes, the mobile nodes are commanded to occupy the cells with the bigger sequence numbers first.

**Figure 16.**
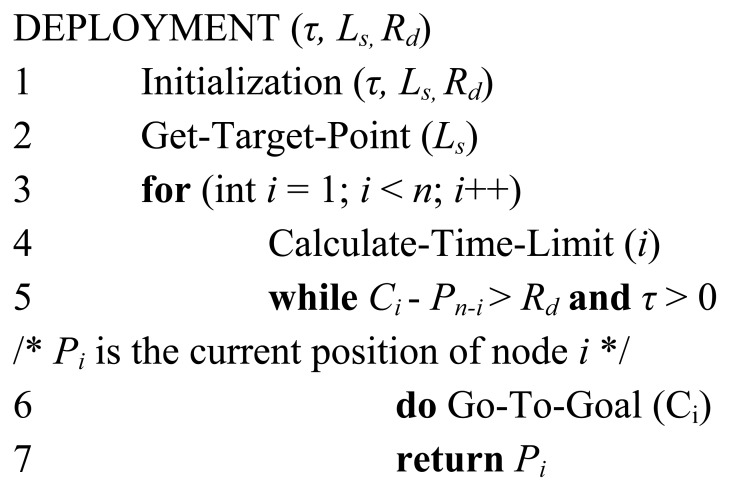
Pseudocodes of the algorithm for autonomous deployment.

Figure 16 shows the pseudocodes of the algorithm for autonomous deployment. The *Rc* value used in this experiment is about 20 meters, which is much longer than the physical size of the testbed. Every node communicates with the base station by only one hop. So the influences of communication range are not considered here.

**Figure 17.**
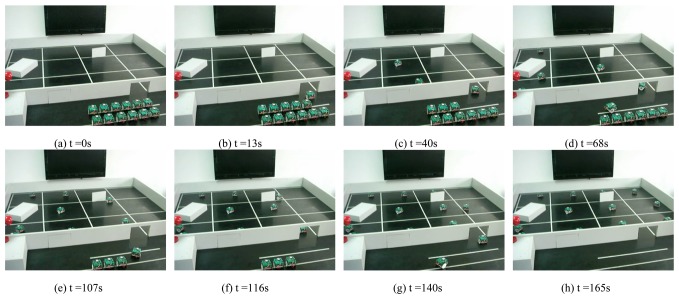
Deployment of a group of twelve mobile sensor nodes.

Before running the deployment routines, we have to assign a timing limit to each mobile sensor node in order to avoid trapping by obstacles. The timing limit of the mobile node to reach *C_i_* is in proportion to the moving speed and the traveling distance to the target point. If the mobile node can not reach *C_i_* before *τ_i_*, then we consider *C_i_* as unreachable and the mobile node stops. The obstacles are randomly distributed in the testbed. The node can autonomously avoid those obstacles on the deployment path by using the two infrared sensors. Here we suppose that there are at least *n* mobile nodes available for deployment. Figure 17 shows the process of the deployment experiment on the tabletop testbed. The target area is divided into 12 identical square cells. The value of *L_s_* is 700 mm and the value of *R_d_* is 100 mm. The position of the entrance and the adjacent start point can be localized by GPS or other general positioning methods. The mobile sensor nodes are put in front of the entrance to start its mission of deployment in turn. The average moving speed of each node is about 60 mm/s. Each node pauses for 3 seconds after every 100 encoder counts. During the pause, the node gets its current position data and routes the data back to the base station outside. The deployment experiment was repeated for several times and the stability of the deployment results was satisfying.

**Figure 18.**
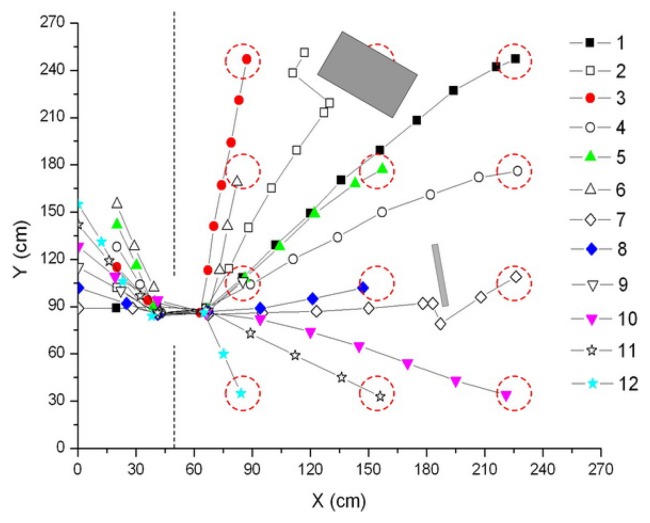
Coverage routes of the mobile nodes during the deployment process.

**Figure 19.**
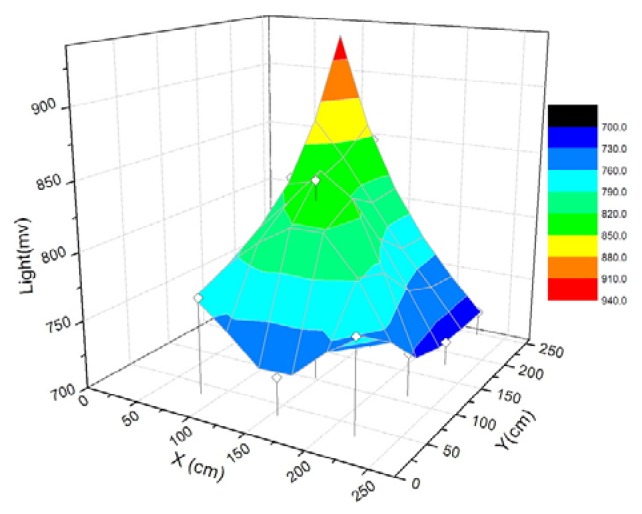
Light gradient measured by the deployed mobile sensor nodes.

The coverage routes measured by the mobile sensor nodes during one of the deployment experiments are shown in Figure 18. One of the target circles can not be reached due to being trapped in the obstacle. The autonomous behaviors of obstacle avoidance will make the mobile node wander to and fro in front of the obstacle. If the mobile node can not reach the target circle after a predefined timing limit, it will stop and terminate the deployment process. Other target circles have all been reached by the corresponding mobile sensor nodes before the timing limit. The light gradient measured by the deployed mobile sensor nodes is shown in Figure 19. The gradient descent from the light source indicates the lighting distribution of the test environment and it can help locate the source when the video data are insufficient or unavailable.

## 5. Conclusions

We have presented a mobile sensor network system for monitoring of unfriendly environments. A wheel-based robotic node architecture for adding mobility to wireless sensor networks has been proposed and some prototype nodes have been implemented. The motion performance of the mobile nodes and the autonomous deployment capability of the proposed mobile sensor network have been tested by some experiments performed on a tabletop testbed. Experimental results show that the proposed mobile sensor network system successfully brings mobile sensing, network self-deployment and event tracking capabilities to wireless sensor networks. Although the system is implemented only on our testbed, it paves a new way for solving the similar problems of sensor network applications in unfriendly environments.
